# Systematic literature review of the economic burden of spinal muscular atrophy and economic evaluations of treatments

**DOI:** 10.1186/s13023-021-01695-7

**Published:** 2021-01-23

**Authors:** Tamara Dangouloff, Camille Botty, Charlotte Beaudart, Laurent Servais, Mickaël Hiligsmann

**Affiliations:** 1 University of Liège, Liège, Belgium; 2 Department of Health Services Research, CAPHRI Care and Public Health Research Institute, Maastricht University, P.O. Box 616, Maastricht, 6200 MD The Netherlands; 3 Department of Paediatrics, Neuromuscular Reference Center Disease, Liège, Belgium; 4 Department of Paediatrics, MDUK Neuromuscular Center, University of Oxford, Oxford, UK

**Keywords:** Burden, Cost, Cost-effectiveness, Economic, ICER, Nusinersen, Spinal muscular atrophy, Onasemnogene abeparvovec

## Abstract

**Background:**

Spinal muscular atrophy (SMA) is a rare and devastating condition for which new disease-modifying treatments have recently been approved. Given the increasing importance of economic considerations in healthcare decision-making, this review summarizes the studies assessing the cost of SMA and economic evaluations of treatments. A systematic review of the literature in PubMed and Scopus up to 15 September 2020 was conducted according to PRISMA guidelines.

**Results:**

Nine studies reporting the annual cost of care of patients with SMA and six evaluations of the cost-effectiveness of SMA treatments were identified. The average annual cost of SMA1, the most frequent and severe form in which symptoms appear before the age of 6 months were similar according to the different studies, ranged from $75,047 to $196,429 per year. The yearly costs for the forms of the later-onset form, called SMA2, SMA3, and SMA4, which were usually pooled in estimates of healthcare costs, were more variable, ranging from $27,157 to $82,474. The evaluations of cost-effectiveness of treatment compared nusinersen treatment against standard of care (n = 3), two treatments (nusinersen and onasemnogene abeparvovec) against each other and no drug treatment (n = 1), nusinersen versus onasemnogene abeparvovec (n = 1), and standard of care versus nusinersen with and without newborn screening (n = 1). The incremental cost-effectiveness ratio (ICER) of nusinersen compared to standard of care in SMA1 ranged from $210,095 to $1,150,455 per quality-adjusted life years (QALY) gained and that for onasemnogene abeparvovec ranged from $32,464 to $251,403. For pre-symptomatic patients, the ICER value ranged from $206,409 to $735,519. The ICERs for later-onset forms of SMA (2, 3 and 4) were more diverse ranging from $275,943 to $8,438,049.

**Conclusion:**

This review confirms the substantial cost burden of standard of care for SMA patients and the high cost-effectiveness ratios of the approved drugs at the current price when delivered in post-symptomatic patients. Since few studies have been conducted so far, there is a need for further prospective and independent economic studies in pre- and post-symptomatic patients.

## Background

Spinal muscular atrophy (SMA) is the most common genetic cause of death in children, with an incidence of approximately 1 in 12,000 live births and a prevalence of approximately 1–2 per 100,000 persons [1]. Patients present with loss of muscle strength followed by onset of progressive paralysis including in the respiratory muscles. Clinical phenotypes are grouped into four forms according to disease severity and age of onset. The most severe form, called type I or "Werdnig-Hoffman disease" (SMA1), manifests during the first 6 months of life. Without respiratory assistance, children with SMA1 usually die during the first 2 years of life [2]. Onset of type II or "intermediate" SMA (SMA2) occurs between the ages of 6 and 18 months. Type 2 can be divided into 2a (patients who sit independently) and 2b (patients who have acquired the standing position but cannot walk). Of patients with SMA type 2a 81% and 67.7% survive without permanent ventilation at ages 30 and 50 years, respectively. Survival without permanent ventilation of patients with SMA type 2b is normal at least within the first 60 years of life [3]. The first symptoms of type III or Kugelberg–Welander disease (SMA3) appear after the age of 18 months. The life expectancy of SMA3 patients is not different from that of the general population [3]. Patients with type IV SMA (SMA4) develop symptoms during the second or third decade of life; patients with this form, also known as "adult form" retain the ability to walk. SMA has severe consequences for patients in terms of mobility and quality of life for patients with all forms [4] and in terms of life expectancy for the most severe and most common forms. SMA is a major cause of disability in children and adults [2, 5] and leads to a substantial economic burden.

An increasing number of studies have investigated the economic impact of SMA in terms of quality of life and cost. One recent study [6] systematically reviewed quality of life studies in SMA and concluded that despite heterogeneous results, quality of life is substantially impaired in SMA, mainly due to poor physical health. To the best of our knowledge, no study has yet systematically reviewed the studies assessing the cost of SMA. Given the increasing importance of economic considerations in pricing and reimbursement decisions, it is important to provide an overview of the overall costs and economic consequences of the SMA.

Recently, three disease-modifying drugs have reached patients’ bedsides [7]: The first to be approved by both the FDA in December 2016 and the EMA in June 2017 was nusinersen [8], marketed as Spinraza by Biogen (Cambridge, MA, USA). Onasemnogene abeparvovec [9, 10], marketed as Zolgensma by Novartis (Basel, Switzerland), was approved by the FDA in May 2019 and the EMA in August 2020. The third entry is risdiplam, an oral compound marketed as Evrisdy, developed by F. Hoffmann-La Roche (Basel, Switzerland), PTC Therapeutics (South Plainfield, NJ, USA), and the SMA Foundation, approved by the FDA in August 2020 [11]; the application to the EMA is pending as of October 2020. Each of these treatments has better efficacy when delivered early [12], which has prompted pre-symptomatic trials [13] and newborn screening programs [14, 15]. Economic comparisons of the costs and the outcomes of these options are necessary as policy makers and payers seek to determine their economic values. Economic evaluations also drive reimbursement and pricing decisions. In this study, we systematically review the economic burden of SMA (in terms of costs) and provide an overview and critical appraisal of economic evaluations in SMA.

## Methods

### Literature search

Two literature searches were conducted using Medline (PubMed) and Scopus (Elsevier) following the PRISMA checklist [16]: one for cost studies of SMA and the second for economic evaluations in the field of SMA. We searched for original, full-text articles reporting costs or economic evaluations of SMA published after January 1, 1998. To identify relevant articles, Medical Subject Headings (Mesh terms) (indexed on Pubmed) and key terms regarding SMA (i.e., “spinal muscular atrophy” OR “Werdnig-Hoffmann” OR “Kugelberg-Welander”) were combined with key terms for costs and economic evaluation. The details of the search strategy are shown schematically in Additional files 1 and 2. In the search for cost studies, the following terms were used: "cost of illness", "price", "pricing", "cost", "costing", "costly", costed", "or healthcare cost". In the search for economic evaluation studies, the following terms were used: “economic”, “health economic”, “cost-effectiveness”, “cost effective”, “healthcare cost”, “health-allocation”, “health-utilization”, “cost-utility”, “cost–benefit analysis”, “cost analysis”, or “economic impact”. Identified articles were manually searched to identify additional articles of relevance. The literature search was last updated on September 15, 2020.

### Selection of studies

Two researchers (TD, CB) first screened titles and abstracts independently for eligibility and then evaluated the full text. To be included, the articles had to be published original research, in English or French, and had to report on cost or economic evaluation in SMA. Economic evaluations were included if they compared both costs and outcomes (e.g., in quality-adjusted life years (QALYs)) between two or more interventions. Articles where SMA was not specifically studied (some articles cover neuromuscular diseases broadly without specific analysis of SMA) and articles where the cost of only a single specific dimension (e.g., ventilation) was reported were excluded. The two reviewers compared their findings, and a list of studies for full-text screening was created. The reasons for article exclusion were recorded, and potential disagreements were specified to be resolved by consensus or, if necessary, with the involvement of a third investigator (MH).

To assess the quality of the economic evaluation, the Consensus on Health Economics Checklist-extended (CHEC-extended) was used [17]. This checklist is an extension of the original CHEC checklist that includes questions about model-based economic evaluations [18, 19]. To limit the possibility of biased results, two reviewers (ChB and TD) independently reviewed the quality appraisal of the included studies. Possible differences in scoring were discussed until consensus was reached. To calculate an overall quality score for each article based on the CHEC-extended checklist, each time a “Yes” was scored, 1 point was allocated, and each time “suboptimal” was scored, 0.5 points were allocated.

### Data extraction and presentation

Studies were thus classified as reporting costs or economic evaluation. Study characteristics related to publication (authors, year of publication, journal name) and study design (country, sample size, population age and gender) were first extracted. For cost studies, we further extracted type of costs, year of costing, time horizon, estimation method, and primary and secondary results. For economic evaluations we extracted type of economic evaluation, perspective, year of costing, time horizon, intervention, comparator, method (trial-based or model-based), outcomes used, results base case, results sensitivity analyses, and funding source. The incremental cost-effectiveness ratio (ICER) is defined as the difference between an alternative and the comparator in terms of costs, divided by their differences in outcomes. The ICER representing the additional cost per QALY gained due to the intervention is then compared to a cost-effectiveness threshold representing the willingness of the decision-maker to pay.

Costs and ICERs were converted to 2020 US dollars to facilitate comparison (data from the Bureau of Labor Statistics’ consumer Price index obtained in October 2020 was used) [20, 21]. For non-US dollars costs, we first translated cost into US dollars of the same year using the exchange rates in the Organisation for Economic Co-operation and Development database [22] and then converted amounts into 2020 US dollars. Cost data are presented by SMA types. SMA1 is typically defined as a SMA that starts before 6 months of age in infants who do not spontaneously acquire independent sitting position. Three articles [23–25] included in our analysis do not use the current classification and consider only two groups: “early onset” (patients who develop symptoms during the first year of life) and "other" (patients who develop symptoms after 1 year of age). We grouped the "early onset" SMA with SMA1. In doing so, some SMA2 patients were categorized as SMA1.

## Results

### Study selection process

**Fig. 1:**
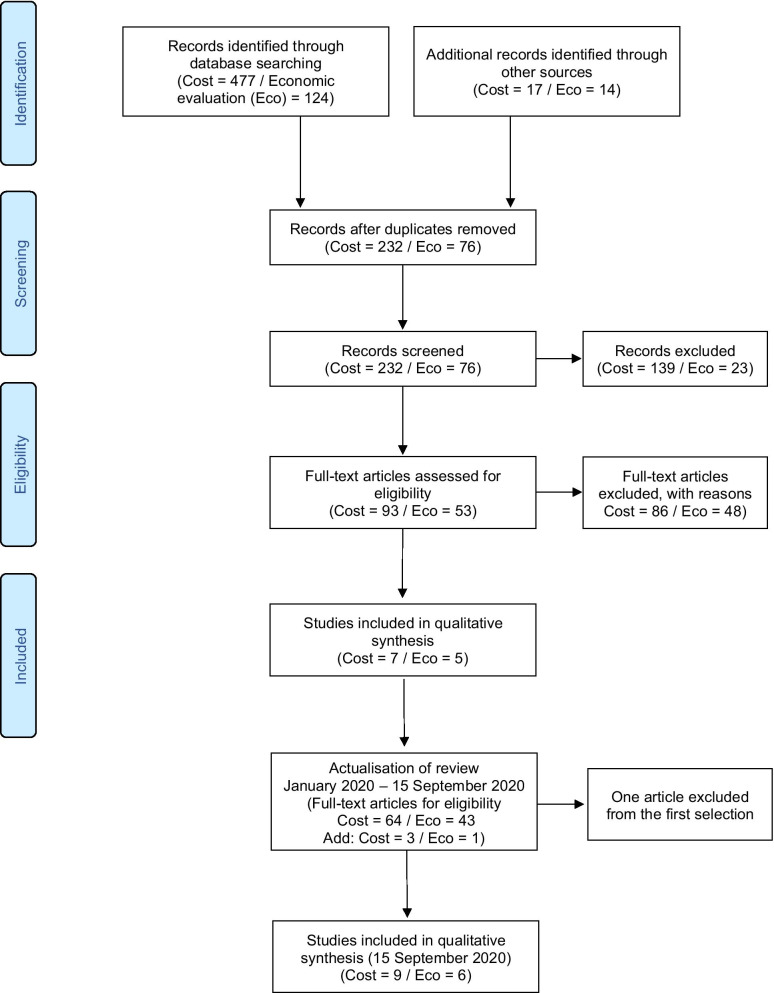
PRISMA flow diagrams of screened articles, updated on 15 September 2020

The initial searches (conducted in December 2019) identified 447 articles that describe cost studies of SMA and 124 economic evaluations of SMA. After removing 232 and 62 duplicates, respectively, and screening by title and abstract, 93 and 76 articles, respectively, were identified for full-text screening. A second search conducted in September 2020 identified 64 references to be screened for costs and 43 for economic evaluation for full-text screening. Of these, nine articles describing the cost of SMA and six describing economic evaluation were included. Figure 1 shows the flow chart based on Preferred Reporting Items for Systematic Reviews and Meta‐Analyses (PRISMA) guidelines [16] used for the identification of these studies.

### Costing studies

Nine cost studies were identified. One study provided cost perception through interviews with seven families [26]. It was excluded because no monetary values were provided. The characteristics of included studies for the cost of SMA are reported in Table 1.

**Table 1:** Overview of literature on cost of SMA

References	Country Year	Sample size	Population age	Type of study	Perspective	Type of cost	Year of costing	Funding
Armstrong et al. [24]	USA 2016	239: 45 < 1 year 194 > 1 year	7.5+/− 6.4	Cross-sectional, retrospective, and prospective	Healthcare costs	Direct healthcare	2003–2012	Conducted by Biogen
Chambers et al. [31]	Australia 2020	40: 4 SMA1 26 SMA2 10 SMA3	SMA1: 2.7 (1–5) SMA2: 9.8 (2–22) SMA3: 6.9 (1–12)	Cross-sectional retrospective	Societal costs	Direct healthcare Direct non-healthcare Indirect	2016–2017	Funded by the Motor Neurone Diseases Research institute of Australia Beryl Bayley
Darba et al. [27]	Spain 2020	396 SMA1, 2 3 and 4		Cross-sectional retrospective	Healthcare costs	Direct healthcare	2014–2016	No
Droege et al. [29]	USA 2019	6526: 349 SMA1 45 SMA1 treated with nusinersen 5728 SMA2, 3, 4 404 SMA2, 3, 4 treated with nusinersen	SMA1: 9.2 months SMA1 nusinersen: 12.2 months SMA others: 30.9 years SMA others nusinersen: 14.8 years	Retrospective	Healthcare costs	Direct healthcare	09/2016–08/2018	Conducted by Avexis
Klug et al. (30)	Germany 2016	189: 12 SMA1 73 SMA2 104 SMA3	< 1 to 73	Cross-sectional retrospective	Healthcare and societal costs	Direct healthcare Direct non-healthcare Indirect	2013	Grant of the Friedrich-Baur-GmbH m’
Lee et al. (25)	USA 2019	229 severe SMA (< 1 year)		Cross-sectional retrospective	Healthcare costs	Direct healthcare	2005–2013	No
Lewin Group (23)	USA 2012	745: 14 early onset SMA 731 SMA other (3–4)	< 1 to 65	Cross-sectional retrospective	Healthcare and societal costs	Direct healthcare Direct non-healthcare Indirect	2008	Conducted by Muscular Dystrophy Association
Lopez-Bastida et al. [61]	Spain 2017	81: 8 SMA1 60 SMA2 13 SMA3	7.22	Cross-sectional retrospective	Healthcare and societal costs	Direct healthcare Direct non-healthcare	2015	Supported by Biogen
Peña-Longobardo et al. [28]	France, German, UK 2020	86: 23 SMA1 45 SMA2 18 SMA 3	6.9	Cross-sectional prospective	Societal costs	Direct healthcare Direct non-healthcare	2015	Supported by Biogen

**Fig. 2:**
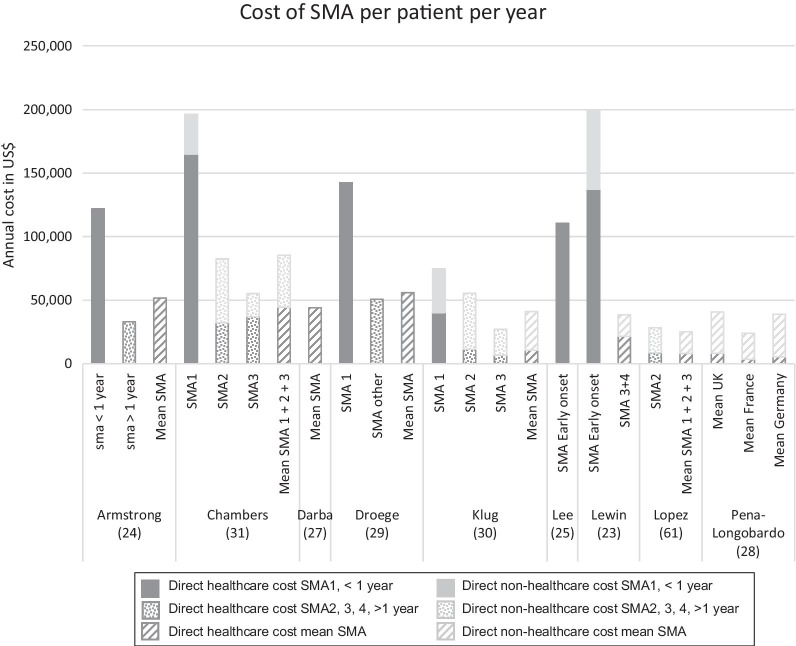
Yearly cost of the illness (US$) in SMA by type

Some studies presented only direct healthcare costs, and others also included direct non-medical costs of the disease (vehicle and home modification, for example). A few studies also estimated indirect costs. Indirect costs were collected through questionnaires submitted to families and captured informal care provided by parents and loss of income of the primary caregiver due to absenteeism from work [35]. Two studies presented costs for all types of SMA together [27, 28]. For the remaining seven articles, costs were classified by type of SMA. With the exception of one study [29] that compared the costs with and without therapy, the other studies reported costs of the disease and are not based on a potential treatment or a comparison of treatment costs. The average annual costs of SMA1 (including early onset and SMA before one year) for the six studies for which these costs were determined, ranged from $75,047 to $196,429 per year [23–25, 29–31]. The costs for the other groups were also variable, ranging from $27,157 [30] to $82,474 [31]. Figure 2 presents the costs by type of SMA.

One study [29] estimated the costs of patients treated with nusinersen compared to those not treated. Total cost per year of a patient with SMA1 decreased significantly from $142,386 without treatment to $95,820 with nusinersen treatment when excluding drug cost. The cost of nusinersen included in these studies varied from $516,896 [35] to $907,665 [29] in the first year, and from $ 258,448 [35] to $457,889 [29] in the second year. For SMA2, 3, and 4 patients, the costs excluding drug costs increased from $50,875 to $79,012 without treatment compared to with treatment. This suggests that nusinersen lowered health care costs, but this should be interpreted with caution as drug costs were not included in the analysis. Comparing total health care costs including drug costs is necessary to provide a fair comparison between active drugs (such as nusinersen and onasemnogene abeparvovec) and standard of care.

### Economic evaluations

Six economic evaluations were identified. The characteristics of included studies are reported in Table 2. Given the heterogeneity between studies, a narrative analysis was conducted.

**Table 2:** Overview of economic evaluation studies of SMA

References	Country	Perspective	Time horizon	Method	Outcomes used	Industry funding
CADTH [34]	Canada 2018	Healthcare payer	SMA1: 25 years SMA2: 50 years SMA3: 80 years	Three Markov models: for SMA1, for SMA2, for SMA3	Life years QALY	No, commissioned by health authorities
ICER [36]	England 2018	Healthcare payer and societal perspective	Two scenarios: 5 years 10 years	Three Markov models: for SMA1, for SMA2 and SMA3, for pre-symptomatic SMA	QALY	No, commissioned by health authorities
Jalali [32]	USA 2020	Societal perspective	30 months	Four Markov models: for untreated patients SMA1, for treated SMA1 identified by symptoms, for untreated patients identified by newborn screening, for nusinersen-treated patients identified by newborn screening	Life Years QALY	No
Malone et al. [33]	USA 2019	Healthcare payer	Lifetime horizon	Markov model comparing nusinersen and Onasemnogene abeparvovec for SMA1	QALY	Avexis
National Center for Pharmaco-economics [37]	Ireland 2017	Societal perspective	Lifetime horizon	Two separate Markov models: for early-onset SMA, for later-onset SMA	QALY	No, commissioned by health authorities
Zuluaga-Sanchez et al. [35]	Sweden 2018	Societal and payer perspective	SMA1: 40 years SMA2: 80 years	Markov model: incremental cost QALY gained and overall survival. Two models: for early-onset SMA, for later-onset SMA	QALY	Biogen

Clinical results for all of the identified studies used data from the following clinical trials:

- Randomized controlled trials:
  o ENDEAR (NCT02193074), which assessed safety and efficacy of nusinersen in SMA1.
  p CHERISH (NCT02292537), which assessed safety and efficacy of nusinersen in SMA2. All studies used QALYs as outcome, and health-state values (or utilities) were derived from this trial
- Non-randomized uncontrolled trials:
  o NURTURE (NCT02386553), that assessed safety and efficacy of nusinersen in pre-symptomatic patients
  p START (NCT03421977), which assessed safety and efficacy of onasemnogene abeparvovec in patients with SMA1.

Each of these four trials showed that treatment statistically and significantly improved motor milestones and resulted in sustained and clinically significant improvements in event-free survival, overall survival, and motor function for children, although follow-up periods were limited. All economic evaluation studies used QALYs as outcome, and health-state values (or utilities) were derived from the CHERISH trial. All studies used trials Endear for motor evolution with nusinersen and one of them [32] used Nurture*.* Vignette studies were also used to obtain utility values in the pediatric and later-onset models [33–35]. Characteristics of these studies by population, intervention, and results are listed in Table 3.

**Table 3:** Overview of the characteristics of the six economic evaluations by population, intervention, and results

References	Population	Intervention/comparator/(including drug prices)	Results (Drugs costs are never included in the analysis)
CADTH [34]	SMA1, 2, 3	Nusinersen versus standard of care Nusinersen: First year: 578,916 US $ Per year after 289,458 US $	For SMA1: Nusinersen led to greater QALYs (gain of 4.80), life years (gain of 4.79), and cost (increase of $3.1 million) for an incremental cost per QALY gained of $665,570 For SMA2: Nusinersen led to greater QALYs (gain of 3.67), life years (gain of 2.18), and cost (increase of $7.6 million) for an incremental cost per QALY gained of $2.1 million For SMA3: Nusinersen led to greater QALYs (gain of 1.56), no difference in life years (gain of 2.18), and an increase in cost ($4.5 million) for an incremental cost per QALY gained of $2.9 million For all three SMA types: The probability that nusinersen was cost effective assuming that the threshold value for a QALY was $300,000 was 0%
ICER [36]	SMA1, 2, 3 and pre-symptomatic	Nusinersen versus standard of care and Onasemnogene abeparvovec versus standard of care Nusinersen: Per year after the first: 396,443 US $ Onasemnogene abeparvovec: 2 million US$	ICER of nusinersen is $709,000 per QALY gained from a healthcare-sector perspective and $687,000 from a modified societal perspective, far exceeding usual cost-effectiveness thresholds For Onasemnogene abeparvovec (at a placeholder price of $2 million) the ICER from a healthcare-sector perspective in patients with symptomatic SMA1 is $243,000 per QALY gained
Jalali [32]	SMA1 and pre-symptomatic	Standard of care compared to Nusinersen with and without newborn screening Nusinersen: First year: 776,000 US $ Per year after: 388,000 US $	Compared with no screening and no treatment, the ICER for nusinersen with screening was $330 558 per event-free life year saved The ICER for nusinersen treatment without screening was $508,481 per event-free life year saved For nusinersen with screening to be cost-effective at a willingness-to-pay (WTP) threshold of $50,000 per event-free LY saved, the price would need to be $23,361 per dose, less than one-fifth its current price of $125,000 Preliminary data from the NURTURE trial indicated an 85.7% improvement in expected LYs saved compared with our base results -In probabilistic sensitivity analysis, nusinersen and screening was a preferred strategy 93% of the time at a $500,000 WTP threshold
Malone et al. [33]	SMA1 patients with 2 copies of *SMN2*	Onasemnogene abeparvovec was compared to nusinersen. Nusinersen: First year: 776,000 US $ Per year after: 388,000 US $ Onasemnogene abeparvovec: between 2,5 and 5 million of US$	Expected survival (undiscounted) over a lifetime predicted by the model was 37.20 life years for Onasemnogene abeparvovec and 9.68 life years for nusinersen (discounted QALYs, 15.65 and 5.29, respectively) Using a potential Onasemnogene abeparvovec price range ($2.5–5.0 M/treatment), the average lifetime cost/patient was $4.2–6.6 M for Onasemnogene abeparvovec and $6.3 M for nusinersen The ICER range was (-$203,072) to $31,379 per QALY gained for Onasemnogene abeparvovec versus nusinersen, indicating that Onasemnogene abeparvovec was cost-effective when priced at ≤ $5 M per treatment
National Center for Pharmaco-economics [37]	SMA1, 2, 3, 4	Nusinersen versus standard of care First year: 681,421 US$ Per year after: 341,105 US$	Nusinersen cannot be considered cost-effective at current price A tenfold reduction in the price of nusinersen for the treatment of infantile SMA is required to produce an ICER approaching the € 45,000/QALY threshold For later-onset SMA, nusinersen is less cost-effective and a 20-fold price reduction results in an ICER just under € 100,000/QALY The 5-year net budget impact for Ireland is estimated at €37.88 million
Zuluaga-Sanchez et al [35]	SMA1, 2	Nusinersen versus standard of care First year: 516,896 US$ Per year after: 258,448 US$	For SMA1: Nusinersen resulted in 3.86 patient incremental QALYs Nusinersen resulted in 0.02 caregiver incremental QALYs Nusinersen incremental cost was $280,000 over standard of care ICER for nusinersen (including caregiver QALYs) of $544,000 per QALY gained For SMA2: Nusinersen resulted in 9.54 patient incremental QALYs Nusinersen resulted in 2.39 caregiver incremental QALYs Nusinersen incremental cost of $3.6 million over standard of care ICER for nusinersen (including caregiver QALYs) of $308,000 per QALY gained

All studies used a decision-analytic model, specifically the Markov model. The models were built on different health states: the motor function milestones achieved, the need for permanent ventilation, and the time to death. For the motor function, the CHOP INTEND or HFMSE scales were used as a reference. The baseline scores were those before the start of treatment. The studies assume that motor function does not improve naturally in SMA patients. These scores were then compared to the scores at the ends of the trials. Patients’ ability to sit and walk was also taken into account. The health states used differed slightly in each study. For example, two studies follow the same model and used the same health states that were used for the submission of the file for drug reimbursement [34, 35]: capacity to sit without support, to stand with assistance, to walk with assistance, to stand unaided, and to walk unaided. Ventilation was also studied with patients categorized as completely autonomous, with need for partial ventilation (during the night), or with permanent ventilation.

### Quality of the economic evaluations

Critical appraisal of the quality of the studies was assessed with the CHEC-extended. The results are available in Table 4. The studies are most often non-qualitative, do not generalize the results to another dimension or pathology, and do not approach the question from an ethical point of view. Most approach the sensitivity of the results only in a probabilistic and non-deterministic way. For half of the studies, the sources of cost data were not clearly identified. Apart from these shortcomings, the studies had scores showing high quality.

**Table 4:** Critical appraisal of the quality of the economic evaluation (CHEC-extended scores)

Authors	CADTH [34]	ICER [36]	Jalali [32]	Malone [33]	NCP [37]	Zuluaga-Sanchez [35]
1. Is the study population clearly described?	1	1	1	1	1	1
2. Are competing alternatives clearly described?	1	1	1	1	0.5	1
3. Is a well-defined research question posed in answerable form?	1	1	1	1	1	1
4. Is the economic study design appropriate to the stated objective?	1	1	1	1	1	1
5. Is the chosen time horizon appropriate in order to include relevant costs and consequences?	1	1	1	1	1	1
6. Is the actual perspective chosen appropriate?	0.5	1	1	0.5	1	1
7. Are all important and relevant costs for each alternative identified?	0	1	1	1	1	1
8. Are all costs measured appropriately in physical units?	0	1	1	1	0	1
9. Are costs valued appropriately?	0	0.5	1	1	0	1
10. Are all important and relevant outcomes for each alternative identified?	1	1	1	1	1	1
11. Are all outcomes measured appropriately?	1	1	1	1	1	1
12. Are outcomes valued appropriately?	1	1	0	1	1	1
13. Is an incremental analysis of costs and outcomes of alternatives performed?	1	1	1	1	1	1
14. Are all future costs and outcomes discounted appropriately?	1	1	0.5	0.5	0	1
15. Are all important variables, whose values are uncertain, appropriately subjected to sensitivity analysis?	0.5	0.5	0.5	0.5	0.5	0.5
16. Do the conclusions follow from the data reported?	1	1	1	1	1	1
17. Does the study discuss the generalizability of the results to other settings and patient/client groups?	0	1	0	0	0	0.5
18. Does the article indicate that there is no potential conflict of interest of study researcher(s) and funder(s)?	1	1	1	1	1	1
19. Are ethical and distributional issues discussed appropriately?	0	0	0.5	0	0	0
Total %	68.4%	89.5%	81.6%	81.6%	68.4%	89.5%

Results of economic evaluation.

**Fig. 3:**
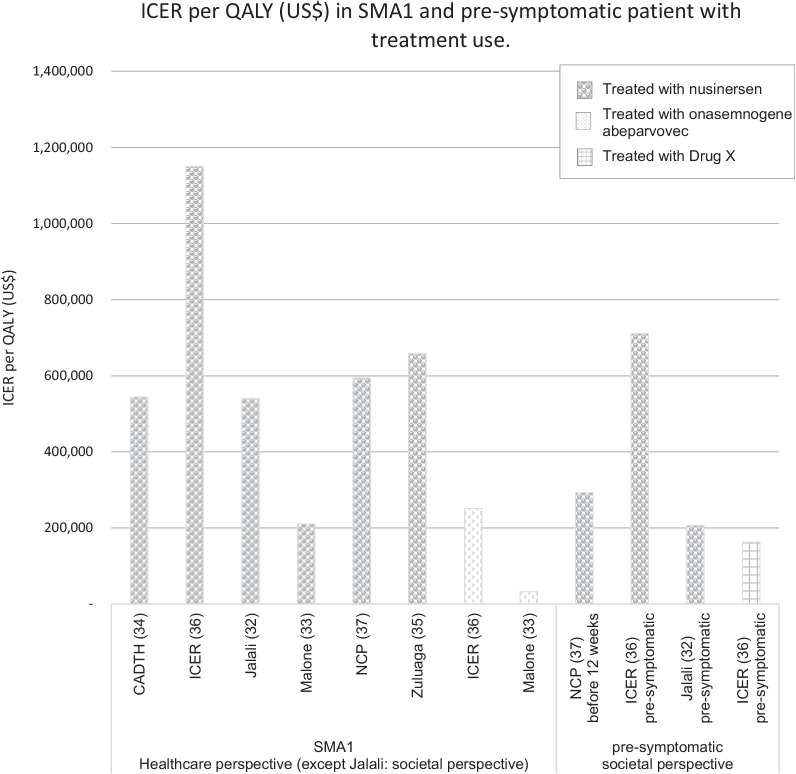
ICER per QALY (US$) in SMA1 and pre-symptomatic patient with treatment use

Of the 6 comparisons, five compared a drug treatment to standard of care (no treatment). Only one study compares the two treatments, i.e., onasemnogene abeparvovec compared to nusinersen [33]. In this study, at the price of $5 million the ICER of onasemnogene abeparvovec compared to nusinersen was $32,464 per QALY (i.e., the total cost of onasemnogene abeparvovec was greater and effectiveness higher than nusinersen). The ICER per QALY gained upon treatment of SMA1 patients with nusinersen compared to standard of care ranged from $210,095 [33] to $1,150,455 [36]; for treatment with Onasemnogene abeparvovec the range was from $32,464 [33] to $251,403 [36]. The ICER per QALY gained with nusinersen versus standard of care for SMA1 patients treated before the age of 12 weeks or pre-symptomatically was $206,409 [32], $293,447 [37] and $710,758 [36]. Figure 3 summarizes the findings from each study.

In the three studies that evaluated ICERs from both societal and healthcare perspectives [35–37], the results for patients treated pre-symptomatically showed a lower ICER from the societal perspective compared to the healthcare payer perspective: for example, $293,447 versus $564,657 for treatment with nusinersen [37]. A similar finding was reported in patients with later-onset SMA: $1,228,612 versus $2,496,442 [37]. No difference was, however, observed between ICERs as evaluated from a societal or healthcare payer perspective in SMA1 treated by nusinersen: ($670,756 for societal perspective versus $658,578 for healthcare payer) [35].

In one study that evaluated the ICER in pre-symptomatic patients [36], the authors assumed that in absence of treatment 60% of patients would develop SMA1, 30% would developed SMA2, and 10% SMA3. This distribution is slightly different from that reported in a recent literature review [1] that found 20–30% of subjects would develop SMA2 and 10–20% would develop SMA3. This discrepancy may have affected the results of the original studies. Scenario analyses were also conducted for a hypothetical drug therapy ("drug X") that had the unique costs of Onasemnogene abeparvovec with QALYs associated with nusinersen in patients with pre-symptomatic SMA. Given the uncertainty in the long-term prognosis of the pre-symptomatic population, scenario analyses for Drug X were performed assuming lower survival. In this study, the cost of the nusinersen treatment was assumed to be $776,000 for the first year and $388,000 per year for the following years [32].

**Fig. 4:**
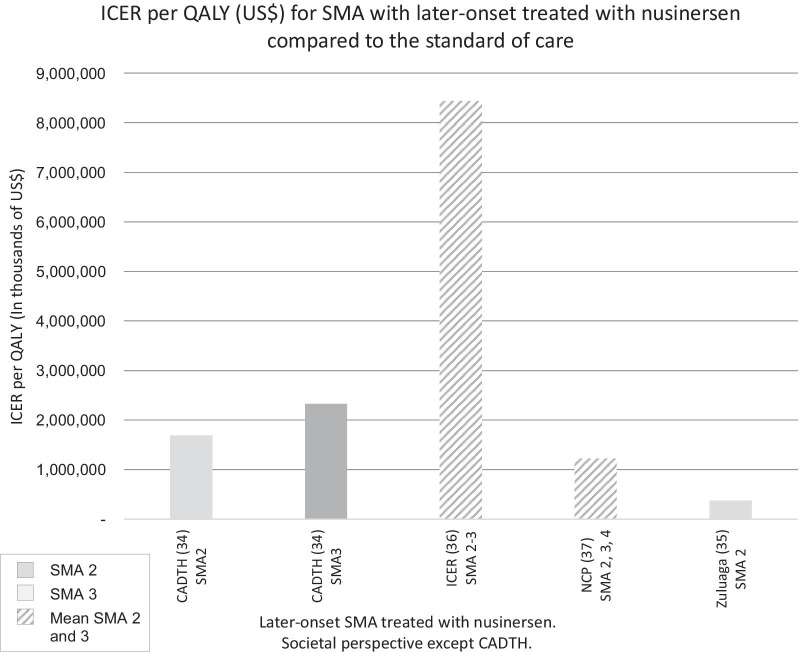
ICER per QALY (US$) for SMA with later-onset treated with nusinersen compared to the standard of care

ICER per QALY in SMA1 for the use of nusinersen or Onasemnogene abeparvovec compared to standard of care. Values are shown for all SMA1 patients and for SMA1 treated before 12 weeks, which is usually pre-symptomatically, with nusinersen or drug X. Drug X is hypothetical and has the costs associated with Onasemnogene abeparvovec and efficacy associated with nusinersen. Figure 4 shows the ICER per QALY for SMA types with later-onset treated with nusinersen compared to the standard of care from a societal perspective. The ICERs for these forms of SMA [2, 3 and 4] varied considerably depending on both the study and the type of SMA from $379,011 [35] to $8,438,049 [36].

## Discussion

This study systematically reviewed all cost studies (n = 9) and economic evaluations (n = 6) of SMA care and treatment up to September 2020. Cost studies quantify the substantial cost of SMA, particularly of SMA1, which has annual costs estimated to range from $75,047 to $196,429 per year, exclusive of drug costs. For other SMA types, a much broader range of costs were observed. The broad range is probably related to the fact that SMA2, SMA3, and SMA4 were considered as a single group, yet their health resource consumptions are very different: Patients with SMA2 are wheelchair ambulant, whereas SMA4 patients remain ambulant. Most SMA2 patients eventually develop restrictive pulmonary syndrome, leading to frequent infections and need of chronic respiratory support; this syndrome is observed much less frequently in SMA3 patients and rarely in SMA4 patients [4]. Another reason for this discrepancy could be the countries in which these different studies were conducted, and the methodologies used. The two studies that reported the highest costs were conducted in European countries, and the others were conducted in the US. In terms of methodology, the two studies that reported the highest costs took indirect cost of illness into account [23, 31].

The yearly cost of SMA1 is significantly higher than those of SMA2 and SMA3. Because life expectancy is shorter in SMA1 [38–40], the total lifetime cost and budgetary impact may be lower than for SMA 2 and SMA3. These huge costs for the later-onset forms are exclusive of new disease-modifying drugs. Nusinersen, the first FDA-approved medication costed from $516,896 [35] to $907,665 [29] in the first year, and from $ 258,448 [35] to $457,889 [29] in the second year. (Different prices estimated between 2017 and 2020, in the US and Europe). Onasemnogene abeparvovec, the second FDA approved drug is considered to be the most expensive drug of the world and is priced at $2.1 million in the US for a single injection. Nusinersen has been approved for use for all types of SMA, yet pivotal studies were conducted only in SMA1 patients younger than 7 months and in SMA2 patient younger than 9 years [8, 41]. Two studies with data from patients followed outside clinical trials confirmed this efficacy in patients from 8 months to 9 years. Patients were followed for 6 months in the first study [42]. In the second study, patients ranged in age from 2.5 years to 8.5 years and were followed for 14 months [43]. Progression was more limited in older than in younger patients.

In one study funded by a pharmaceutical companies [29], a substantial yearly decrease of healthcare costs of $45,000 per patient was observed after nusinersen treatment. However, this decrease was not inclusive of the cost of treatment. A yearly cost comparison in SMA1 patients on treatment or on best standard of care but without treatment is only partially relevant. Indeed, survival of SMA1 patients without treatment and without mechanical ventilation beyond the age of two years is rare [3], which limits the budget impact of these patients. Since treated patients survive longer, the total lifetime cost, and thus the budget impact, of these patients could be much larger than for those on standard of care therapy [44].

Although it has been hypothesized that treated patients are those who have very severe symptoms who would have very high healthcare costs if left untreated, there is currently no data to support this hypothesis. Those who did not benefit from treatment, and whose costs were collected for the study retrospectively from a database that captures prescriptions claims, medical utilization, and costs, would be those who did not urgently require treatment. These are patients for whom the healthcare costs are consequently lower than for the patients on treatment. This suggests that the cost of the disease for people with later-onset SMA who receive treatment is greater than for those given standard of care. As these are two different populations, cost analysis should treat them differently. Treatment of prior to symptom onset has been shown to be more effective than is treatment after symptoms develop [13]. Pre-symptomatic treatment may result in a greater reduction in SMA costs, as shown in the economic evaluation reported by Jalali et al. [32]. This type of analysis could have a major impact on the launch of newborn screening programs.

It should be noted that these costs are not surprising in the context of rare diseases, even if the treatment for SMA is exceptionally high (as a reminder: the cost for nusinersen was estimated in 2020 at $776,000 for the first year, and $388,000 per year for the years after). For example, the infantile form of Pompe disease results in annual costs of $41,667 for standard of care, whereas treatments are estimated at $287,870 annually [45]. The burden of cystic fibrosis, a severe pulmonary disease characterized by frequent pulmonary infections and median survival of about 50 years, can to a certain extend be compared to the burden of SMA2. The estimated yearly cost of cystic fibrosis in 2016 is $131,879 for standard of care therapy including respiratory management and nutrition management [46]. Since 2016, new treatments have been approved that cost $300,000 per year [47]. Duchenne muscular dystrophy, whose level of disability can be compared to SMA3, has annual costs for standard of care around $50,000 [48, 49]. Altogether, the reported cost for SMA benchmarks closely with the costs of other rare diseases that present with a similar level of disability.

Economic evaluations of new drug treatments for SMA have been conducted, but these studies are based on very few trials that included a limited number of patients followed for a very limited period of time. For these reasons, extrapolations were made. The medical data concerning the evolution of treated patients, as well as the costs generated by the disease in treated patients, are under-studied. For example, the QALYs used for the ICERs for nusinersen in SMA2 patients all come from a single study (CHERISH). Caution should also be exercised when comparing data between treatments, as the populations studied are not always comparable. Indeed, disease duration has been consistently shown to be the main predictor of treatment efficacy [12], and disease duration significantly differed between the two therapeutics trials conducted in patients with SMA1 (ENDEAR and START). Motor baseline levels, which has also been shown a predictive factor [42, 50], also differed between the two studies. Another limitation resides in the fact that trials did not collect utility values from patients or caregivers. Only vignettes were used to consider utility; these are not qualitative and are highly variable (e.g., the same health status was assessed at—0.13 to 0.73 [35]), and no single study appeared to capture the burden of disease in all the health states of interest. A final limitation is that all studies conducted to date have been retrospective. Long-term prospective follow-up of patients is needed to capture costs and outcomes for all types of SMA.

In addition, only one economic evaluation has examined specifically the cost-effectiveness of newborn screening for SMA. Given the increase in screening programs and their potential value [14, 15, 51, 52], such economic evaluations are needed. Recent data have suggested that patients treated before symptom onset will have a different future than children treated after symptoms appear [13]. If these patients have much less severe or no disabilities, the economics of treatment will be considerably impacted. Indeed, the cost of the treatment is the same whether it is provided before or after the first symptoms. The difference will be related to the cost of the associated handicap, which will be nil or almost non-existent in pre-symptomatic patients.

Due to the extremely high drug costs, the ICER values for the currently approved SMA therapies are high, and, therefore, treatments are not cost effective. It is important to acknowledge that discounted prices for SMA drugs are confidentially negotiating with payers. Cost-effectiveness analyses based on official prices may therefore overestimate the real cost-effectiveness of SMA drugs. One of the studies [32] provides recommendations for alternative prices based on a sensitivity analysis. Using data from the ENDEAR trial, this analysis suggests that to achieve a willingness to pay threshold of $50,000 per life years saved, a nusinersen dosage price of 19% of the current price would be required. With the arrival on the market of three therapies, prices should tend to decrease, which could then make the prices more acceptable. In addition, these new therapies are expected to become the standard of care, and subsequent economic evaluations will need to include drug therapy as a comparator.

Despite high costs, the approved drugs have been granted reimbursement in several countries. In the domain of rare diseases, the small number of patients makes drug development economically challenging. For example, drugs for treatment of Duchenne muscular dystrophy, which results in costs comparable to SMA2 and SMA3, is associated with ICERs ranging from $944,975 to $2,341,474 [53]. Treatments for Fabry, Gaucher, and Pompe diseases range from $283,000 to $3,485,000, from $46,000 to $459,100, and from $162,800 to $1,108,050, respectively [54]. It is becoming accepted that in these types of conditions, the budget impact should be weighed more heavily than the rough ICER value. Since the frequency of the disease is very low, the budget impact is low despite high costs. Therefore, criteria other than cost-effectiveness are important for decision makers, especially for orphan drugs. Value frameworks have been proposed specifically for these rare and debilitating conditions. Garrison et al. have designed a framework value with SMA as an example. These authors suggest the importance of the “real option value”, the “value of hope”, and the "value of knowledge" [55]. Health equity (related to severity of disease), caregiver burden, and family spillovers (in terms of the negative effect on the well-being of family members) are also important in these situations [56].

As treatments for rare diseases are unlikely to be cost effective given their high prices, additional criteria are already being used to inform reimbursement decisions in some countries. One relevant study analyzed use of public funds for orphan drugs in five European countries from the decision-maker's point of view [57]. Another study was conducted in Italy from the patient's point of view focusing on two diseases, cystic fibrosis and hemophilia; it also quantified individual preferences [58]. The two studies concluded that the important factors in the decision-to-pay process are the cost of treatment, the improvement in health of patients, and the value for money. The severity of the disease and the availability of alternative treatments should also be considered but are less important. Furthermore, the technical experts interviewed pointed out that an onset of symptoms in early childhood, diagnosis delay, and treatment side effects should also be considered as important social values. As several criteria are relevant, a multi-criteria decision analysis can constitute a valuable solution for decision-making. It allows the influence of each criterion on the decision and relative importance to be defined, going beyond the simple QALY analysis [57, 58].

This literature review has some limitations. First, only two databases (Medline and Scopus) were searched. The work of Sassi et al. [59] showed that by using only Medline, with appropriate search strategies, researchers can significantly reduce the number of irrelevant references retrieved by their electronic searches that require exclusion by manual selection. They point out that by not using Embase, there is a risk of losing some references compared to Medline, but that Embase does not include a large number of references. These authors conclude that manual searches and searches in databases other than Medline for reviewing economic evaluations have limited incremental return, so that Medline could be considered as the primary source. Nevertheless, we also investigated Scopus, in order to be as thorough as possible.

Second, we limited our search to original articles; conference proceedings were not included. It is likely that data presented at conferences on neuromuscular diseases or SMA will be published soon, as the SMA world is in a period of upheaval given that the recent approvals of effective therapies. Nevertheless, decisions on pricing are being made today on the basis of publicly data available. One of the studies we relied upon was itself a reanalysis and additional limitations were noted: Patient conditions reported are relative (stabilizing, improving, worsening) instead of absolute and were relative to individual patient's baseline conditions and not to motor scale numbers. With respect to clinical trial design, patients who participate in the trials are only a sample of the patient population, particularly in terms of age, and cannot be used as a projection to the entire patient population [34]. A final limitation is that studies funded by the pharmaceutical industry showed lower ICERs. Although the number of studies is too limited to make reliable comparison between industry-sponsored and non-industry sponsored economic evaluations, and the fact that no relationship was observed in other diseases [60], this remains a potential study publication bias as pharmaceutical companies could tend to present most favorable results. Despite the scarcity of economic evaluations of SMA, these few published studies will be central for health authorities who will use these data to drive policy choices. It therefore is important to also consider from research from independent institutes or unsubsidized academic groups.

## Conclusions

In conclusion, this literature review revealed the substantial cost burden of SMA and the high ratio of cost effectiveness of the approved drugs at the current price when delivered in post-symptomatic patients. Few studies evaluating cost and economic benefits of therapy have been conducted so far, and there is a need for further prospective and independent economic studies, in patients treated after symptom onset and in patients who are benefiting from pre-symptomatic treatment.

## Supplementary Information

**Additional file 1**. List of words used for the search

**Additional file 2**. Strategy for search of MEDLINE Ovid

## Data Availability

Data sharing is not applicable to this article as no datasets were generated or analyzed during the current study.

## Abbreviations

EMA: European Medicines Agency
FDA: Food and Drug Administration
ICER: Incremental Cost-Effectiveness Ratio
QALY: Quality-Adjusted Life Years
SMA: Spinal Muscular Atrophy
