# Supplementary material for: Systematic literature review of the economic burden of spinal muscular atrophy and economic evaluations of treatments
Source: Orphanet J Rare Dis. 2021 Jan 23;16:47. doi: 10.1186/s13023-021-01695-7 (PMC7824917; doi:10.1186/s13023-021-01695-7)
Supplement: Supplementary file 1 — Additional file 1. List of words used for the search [file 13023_2021_1695_MOESM1_ESM.pdf]

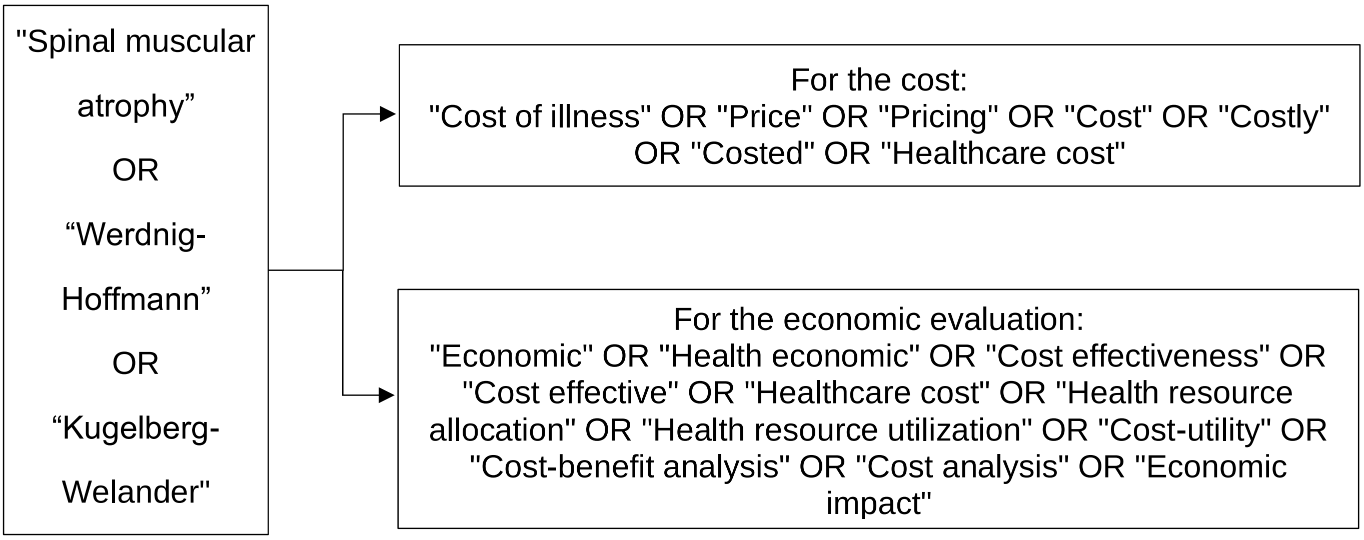
"Spinal muscular atrophy" OR "Werdnig-Hoffmann" OR "Kugelberg-Welander"
For the cost:
"Cost of illness" OR "Price" OR "Pricing" OR "Cost" OR "Costly" OR "Costed" OR "Healthcare cost"
For the economic evaluation:
"Economic" OR "Health economic" OR "Cost effectiveness" OR "Cost effective" OR "Healthcare cost" OR "Health resource allocation" OR "Health resource utilization" OR "Cost-utility" OR "Cost-benefit analysis" OR "Cost analysis" OR "Economic impact"
