# Supplementary material for: Systematic literature review of the economic burden of spinal muscular atrophy and economic evaluations of treatments
Source: Orphanet J Rare Dis. 2021 Jan 23;16:47. doi: 10.1186/s13023-021-01695-7 (PMC7824917; doi:10.1186/s13023-021-01695-7)
Supplement: Supplementary file 2 — Additional file 2. Strategy for search of MEDLINE Ovid [file 13023_2021_1695_MOESM2_ESM.pdf]

Search strategy: Strategy for search of MEDLINE Ovid

For SMA

1. exp "Spinal Muscular Atrophies of Childhood"/ or Werdnig-Hoffman.mp. or Muscular Atrophy, Spinal/ or Kugelberg-Welander.mp.
2. (spinal muscular adj3 atroph*).ti,ab,kf.
3. Kugelberg-Welander.ti,ab,kf.
4. Werdnig-Hoffmann.ti,ab,kf.
5. 1 or 2 or 3 or 4

For cost:

6. exp "Costs and Cost Analysis"/
7. cost*.ti,ab,kf.
8. (price* or pricing).ti,ab,kf.
9. econom*.ti,ab,kf.
10. (burden* or coinsurance or deductible* or (saving* adj3 account*) or expenditure* or expense* or out of pocket* or (capital* adj1 expenditure*)).ti,ab,kf.
11. ((hospital* adj3 charge*) or fee* or pharmacoeconom* or debt* or charge* or (rate* adj3 setting)).ti,ab,kf.
12. "Global Burden of Disease"/
13. (expense* or expenditure* or payment* or out-of-pocket or (care adj2 consumption) or cost of illness or budget* or monetary).ti,ab,kf.
14. (((resource* or drug*) adj2 (utili?ation or allocat* or use*)) or ((health or healthcare or direct service* or indirect service* or hospital* or drug*) adj2 (cost or use* or utili?ation or resource* or consumption)) or financial or reimbursement or ((health* or care) adj2 service)).ti,ab,kf.
15. ((burden adj2 (illness or disease* or health*)) or (cost* adj2 analys*) or cost effective or health policy).ti,ab,kf.
16. (qalys or dalys or quality-adjusted life years or disability-adjusted life years).ti,ab,kf.
17. 6 or 7 or 8 or 9 or 10 or 11 or 12 or 13 or 14 or 15 or 16

For economic evaluation:

18. exp Economics/
19. exp "Costs and Cost Analysis"/
20. "health care economics and organizations"/ or "costs and cost analysis"/ or insurance, health, reimbursement/ or prospective payment system/ or financing, personal/ or health care sector/or economics, hospital/ or hospital charges/ or hospital costs/ or economics, medical/ or fees, medical/ or economics, nursing/ or economics, pharmaceutical/ or external debt/ or "Delivery of Health Care"/ or Health Resources/
21. "Quality of Life"/ or Quality-Adjusted Life Years/
22. Models, Economic/ or Cost-Benefit Analysis/ or Health Care Costs/
23. cost*.ti,ab,kf.
24. econom*.ti,ab,kf.
25. cost-effectiveness analysis.mp.
26. 18 or 19 or 20 or 21 or 22 or 23 or 24

Limits:

27. limit to yr="1998 -Current"
28. limit X to (english or french)
29. limit to yr="2020 -Current"

Research for cost studies of SMA: 5 and 17 and 27 and 28
Research for economic evaluation in the field of SMA: 5 and 26 and 27 and 28
Update: 5 and 17 and 28 and 29 / 5 and 26 and 28 and 29
